# Differential Effects of AAV.*BDNF* and AAV.*Ntf3* in the Deafened Adult Guinea Pig Ear

**DOI:** 10.1038/srep08619

**Published:** 2015-03-02

**Authors:** Cameron L. Budenz, Hiu Tung Wong, Donald L. Swiderski, Seiji B. Shibata, Bryan E. Pfingst, Yehoash Raphael

**Affiliations:** 1 Kresge Hearing Research Institute, Department of Otolaryngology - Head and Neck Surgery, The University of Michigan, Ann Arbor, MI, USA

## Abstract

Cochlear hair cell loss results in secondary regression of peripheral auditory fibers (PAFs) and loss of spiral ganglion neurons (SGNs). The performance of cochlear implants (CI) in rehabilitating hearing depends on survival of SGNs. Here we compare the effects of adeno-associated virus vectors with neurotrophin gene inserts, AAV.*BDNF* and AAV.*Ntf3*, on guinea pig ears deafened systemically (kanamycin and furosemide) or locally (neomycin). AAV.*BDNF* or AAV.*Ntf3* was delivered to the guinea pig cochlea one week following deafening and ears were assessed morphologically 3 months later. At that time, neurotrophins levels were not significantly elevated in the cochlear fluids, even though in vitro and shorter term in vivo experiments demonstrate robust elevation of neurotrophins with these viral vectors. Nevertheless, animals receiving these vectors exhibited considerable re-growth of PAFs in the basilar membrane area. In systemically deafened animals there was a negative correlation between the presence of differentiated supporting cells and PAFs, suggesting that supporting cells influence the outcome of neurotrophin over-expression aimed at enhancing the cochlear neural substrate. Counts of SGN in Rosenthal's canal indicate that BDNF was more effective than NT-3 in preserving SGNs. The results demonstrate that a transient elevation in neurotrophin levels can sustain the cochlear neural substrate in the long term.

Cochlear implants (CI) restore the hearing sense by directly stimulating remnant auditory neural structures in ears in which hair cells are absent or dysfunctional. CIs have been very successful in restoring the recipient's ability to understand speech in quiet settings. However, they have had more limited benefit in restoring the ability to clearly perceive more complex acoustic stimuli, such as speech in background noise or music. Current CIs typically have 8–22 electrodes, but some studies have shown an effective functional yield of only 4–10 perceptual channels, which is likely due to current spread and the deterioration of auditory neuronal structures^1^,^2^. A greater number of functional perceptual channels are required to optimize speech understanding in noise (up to 20) and music appreciation (32 or more)^1^,^3^,^4^. Advances in CI technology, alone, cannot provide the necessary additional perceptual channels, as the improved technology does not address the issue of compromised auditory neural targets. To address this issue, it is likely that advances in biotherapies will be key to further enhancing CI outcomes. It is through biotherapies, such as the delivery of neurotrophins to the inner ear, that auditory neural substrate survival and health may be improved, thereby increasing the number of perceptual channels rendered by our current CI electrode technology.

Neurotrophins are a family of molecules essential in the development and maintenance of neural systems throughout the body. Neurotrophin-3 (NT-3) and brain derived neurotrophic factor (BDNF) are the two neurotrophins critical for the normal development and function of the peripheral auditory nervous system^5^. Studies using null mutant mice have demonstrated that NT-3 is essential for the normal development of the majority of spiral ganglion neurons (SGNs) and afferent peripheral auditory fibers (PAFs)^6^,^7^, whereas BDNF is critical in the development of primarily vestibular ganglion cells and a minority of afferent PAFs^7^. Additional null mutant mouse studies have suggested that there is a gradient of neurotrophin expression within the cochlea that changes over the course of development. BDNF is initially expressed primarily in the apex of the cochlea, with progressively increased expression in the base later in development^8^. In contrast, NT-3 is primarily expressed in the cochlear base early in development, and expression progresses apically in post-natal and adult mice^8^,^9^. Within the adult inner ear, NT-3 is primarily expressed by inner hair cells and supporting cells within the cochlear apex^9^, and BDNF is primarily expressed within the vestibular system^5^.

In addition to their developmental roles, neurotrophins have also been shown to influence the outcome of trauma to the inner ear. For instance, several laboratories have shown that administration of neurotrophin molecules to the deafened inner ear enhances SGN survival and health^10^,^11^,^12^,^13^,^14^. These and other studies have found a beneficial short-term effect of neurotrophins on the auditory neural structures of the deafened inner ear (See Budenz et al. for a recent review^15^). However, it has remained unclear whether any of these beneficial effects persist over the long-term, and whether one neurotrophin molecule is superior in its ability to enhance the survival and health of remnant auditory neural substrates in the deafened adult inner ear.

In this study we examined long-term (3-month) effects of viral-mediated neurotrophin over-expression in deaf ears. An adeno-associated viral (AAV) vector was utilized to administer either NT-3 (AAV.*Ntf3*) or BDNF (AAV.*BDNF*) to the inner ears of deafened adult guinea pigs. The AAV vector was chosen because it has not been associated with human disease and it has been demonstrated to induce long-term expression of its transgene, two features that increase its potential clinical utility^16^,^17^. We deafened guinea pigs bilaterally either by local administration of neomycin into the basal turn of each cochlea or by systemic administration of kanamycin and furosemide (K&F). One week after deafening, animals were inoculated with an AAV vector with a gene insert for one of the neurotrophins and the extent of PAF re-growth and SGN survival at 3 months was measured. Control groups included the contralateral ear, as well as cochleae that were inoculated with an AAV.Empty vector. The use of two different lesions, one severe enough to uniformly flatten the auditory epithelium (neomycin) and the other sparing patches of supporting cells (K&F), allowed us to assess the correlation between the state of the non-sensory epithelium in the deaf ears and the extent of PAF growth.

## Results

### Safety and long term outcome with adeno-associated viral vectors

An adeno-associated viral vector was utilized in this study to induce expression of either *BDNF* or *Ntf3* in the cochlea. There were no animal deaths attributable to the viral vector. Over the course of the study a total of 5 animals required early euthanization due to deteriorating health. Two of these animals were euthanized 3 days following systemic deafening and prior to inoculation with the viral vector. One animal was euthanized nearly 3 months following viral inoculation, and post-mortem examination demonstrated evidence of pneumonia. Two animals required euthanization within one week following inoculation with the viral vector: one animal had evidence of a suppurative middle ear infection suspected to be related to surgical contamination, and one animal had respiratory distress thought to be related to post-anesthetic complications. The remaining 46 animals recovered well from the procedures (deafening and AAV inoculation) and completed the experiment. Their cochleae did not display signs of active infection.

### AAV activity and the concentration of NT-3 and BDNF in culture media and perilymph

To determine activity of the two AAVs, we performed sets of experiments in vitro and in vivo. First, to determine the outcome of viral vector transduction in the short term, we experimented with cultured cells. Guinea pig fibroblasts were incubated for four hours with AAV.*BDNF* or AAV.*Ntf3*. The vectors were rinsed 4 hours later. Controls included AAV.*GFP* or media collected from cells to which no AAV was added. Media was collected 7 days later and analyzed with ELISA. Average concentration of NT-3 in wells receiving AAV.*Ntf3* vector was 2.21 ng/ml, about 2 times the ELISA standard (positive control) and 130 times the concentration in wells that received AAV.*GFP* or no vector (negative control). The difference between *Ntf3* and negative control wells was highly significant (p = 0.003). The average concentration of BDNF in wells receiving that vector was 4.17 ng/ml, about 4 times the standard and 150 times the concentration in AAV.*GFP* or no vector. The difference between AAV.*BDNF*-receiving and negative control wells also was highly significant (p = 0.001). These results validate the strong activity of the AAV vectors in promoting secretion of their corresponding neurotrophin from transduced cells.

We then performed a short term in vivo experiment to determine the influence of the AAV.*BDNF* vector. We inoculated guinea pig cochleae that were deafened with neomycin and collected tissues 3 weeks later. In these ears, we observed a marked influence of the viral vector on the neuronal network in the area of the basilar membrane, which was devoid of neurons in control ears. The AAV.*BDNF* injected ears showed robust sprouting of nerve fibers into the epithelium of the deaf cochlea (Suppl. Fig. 1).

In experimental animals, we sampled neurotrophin levels in perilymph collected from ears sacrificed three months after the AAV.*Ntf3* and AAV.*BDNF* inoculation. One of five samples from ears of animals that received AAV.*BDNF* had a protein concentration (57 pg/ml), nearly double the detection threshold (24 pg/ml); none of the other four were above that threshold. Four of 9 ears that received AAV.*Ntf3* had protein concentrations above the detection threshold (31.3 pg/ml). Two were only just above this level (39–40 pg/ml), and two were much higher (66.6 and 111.4 pg/ml). The levels of neurotrophins detected may reflect, in part, the technical difficulty of collecting microliter amounts of fluid from the perilymphatic space, as well as indicate that the levels of neurotrophins cannot be reliably sustained over three or more months, possibly due to turnover of the transduced mesothelial cells.

### Outcome of deafening and neurotrophin over-expression in the auditory epithelium

**Figure 1:**
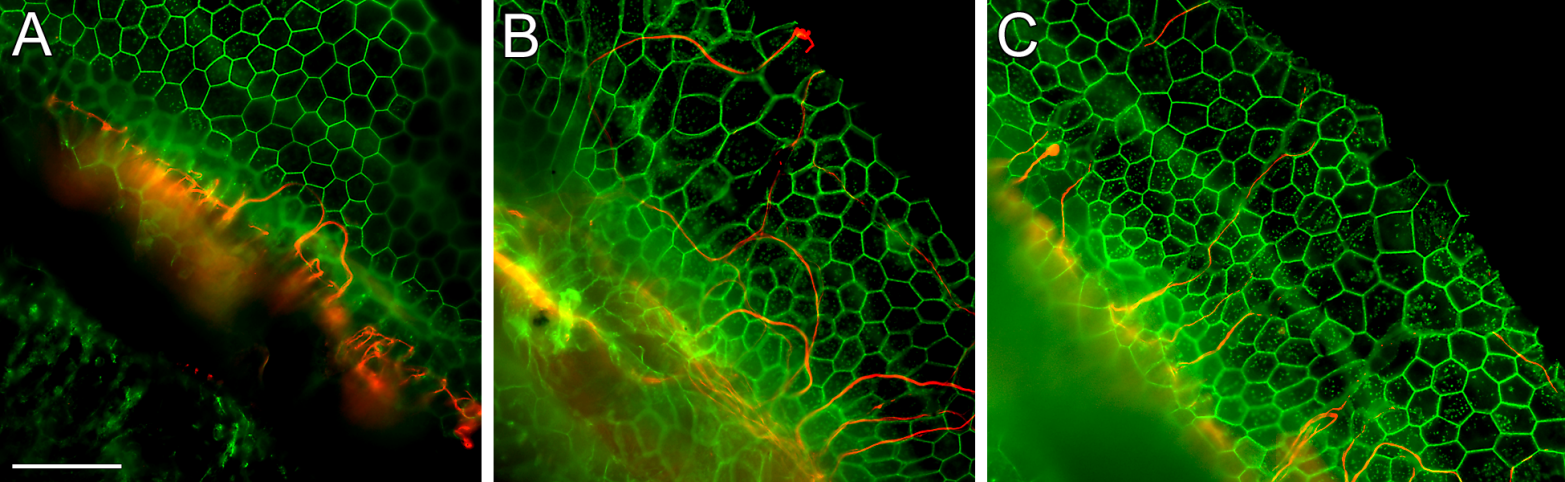
Representative Images of Peripheral Auditory Fiber Re-growth in Neomycin Deafened Ears. Intracochlear neomycin administration resulted in a complete loss of hair cells and supporting cells in the basal turn, forming a flat epithelium. A few looping auditory fibers are seen in the basilar membrane area of un-injected ears (A), whereas significantly more radial fibers are seen in neurotrophin injected ears (B: NT-3 vector, C: BDNF vector). Green = Actin; Red = Neurofilament 200 kDa. Scale Bar = 50 μm.

The effectiveness of the deafening procedure was assessed provisionally in each animal by the Preyer reflex prior to euthanization, and confirmed by the loss of hair cells seen on histological evaluation of the basilar membrane area. Local administration of neomycin into the basal (first) turn of the cochlea had a consistent and severe effect on cochlear morphology (Fig. 1). Neomycin deafened ears had a complete absence of recognizable Deiters and pillar cells in the first turn, as well as complete loss of hair cells (Fig. 1A). Remaining cells appeared as a flat cuboidal epithelium throughout the basal and at least part of the second turn. In the upper second turn and above, Deiters and pillar cells tended to survive; however, no inner or outer hair cells were seen in any cochlear turn (not shown). Inoculating these severely deafened ears with AAV.*Ntf3* (Fig. 1B) or AAV.*BDNF* (Fig. 1C) resulted in growth of PAFs into the flat epithelium that had replaced the organ of Corti in the deaf cochlea.

**Figure 2:**
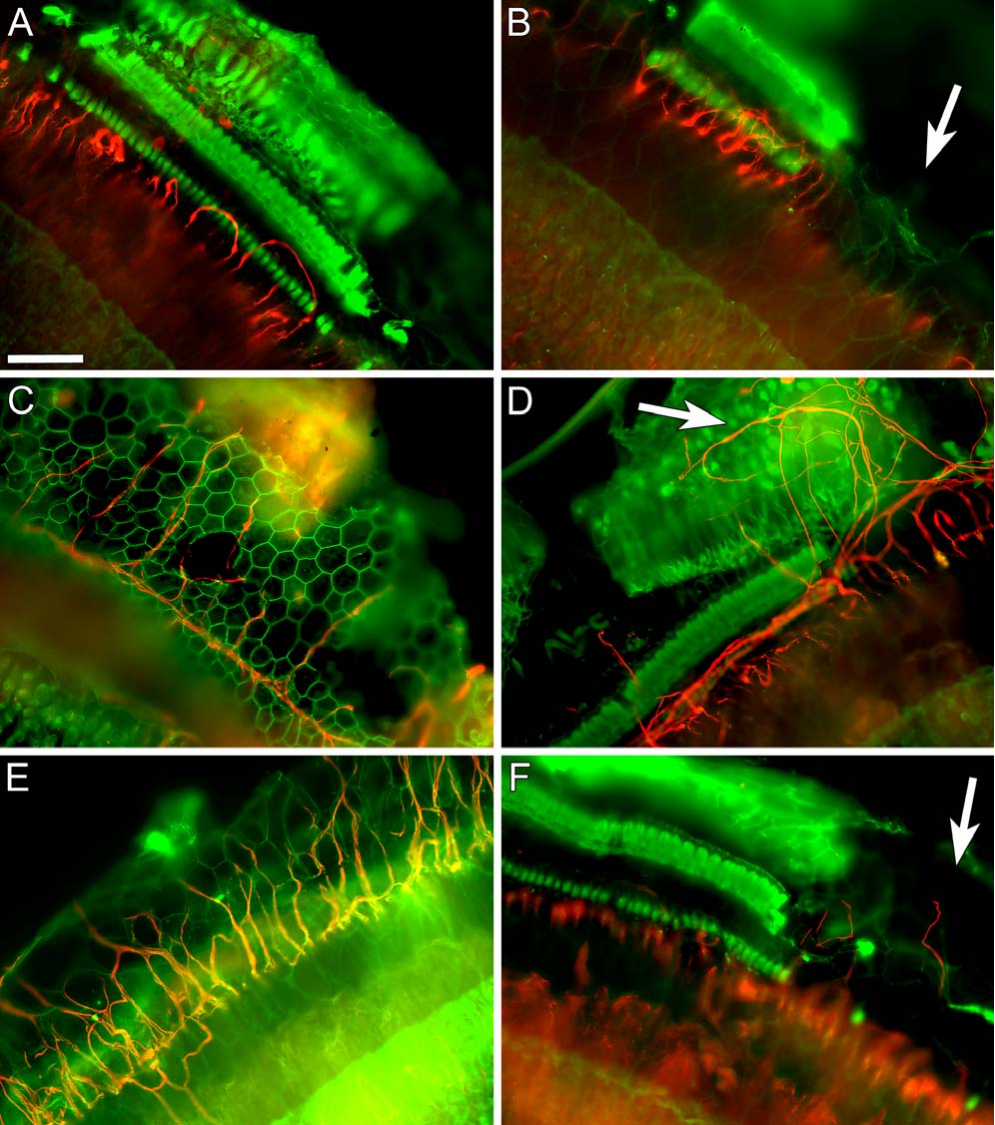
Representative Images of Peripheral Auditory Fiber Re-growth in Systemically Deafened Ears. Systemic administration of K&F resulted in a complete loss of hair cells in the basal turn and patches of surviving supporting cells. The few neural fibers seen in the basilar membrane area of un-injected ears make short loops (A, B). In contrast, neurotrophin vector receiving ears have significantly more neural fibers, which also extend farther from the habenula (C, D: NT-3; E, F: BDNF). Neural fibers did not appear to re-grow in regions of surviving supporting cells (D, F). A prominent re-growth of neural fibers is seen in regions devoid of supporting cells (C, E). Green = Actin; Red = Neurofilament 200 kDa; Scale Bar = 50 μm.

Systemic deafening with K&F had more variable effects on the auditory epithelium and on the ability of viral-mediated neurotrophin over-expression to induce PAF outgrowth (Fig. 2). The basal turn consistently demonstrated a complete loss of hair cells (Fig. 2 A–F); however, Deiters and pillar cells survived in irregular clusters separated by patches of flat epithelium (Fig. 2 A–B). Neurofilament specific staining revealed occasional looping neural fibers close to the habenula perforata but these fibers rarely reached further laterally into the deaf auditory epithelium (Fig. 2 A–B). These looping fibers were not counted as sprouting fibers.

Beginning in the second turn, inner hair cells were intermittently seen, and there was increased survival of supporting cells (not shown). In the third and apical turns of these cochleae many supporting cells and hair cells survived. For this reason, we did not include the upper three turns in our analyses of the systemically deafened ears. In ears of systemically deafened animals that were subsequently injected with the neurotrophin vectors, numerous PAFs were seen extending from the habenula perforata and reaching into the auditory epithelium (Fig. 2, C–D: NT-3; E–F: BDNF). PAFs were numerous and appeared to be evenly distributed in regions where the epithelium was flat (Figs. 2 C and E), whereas PAF were few and widely scattered in regions with surviving supporting cells (Figs. 2D and F).

**Figure 3:**
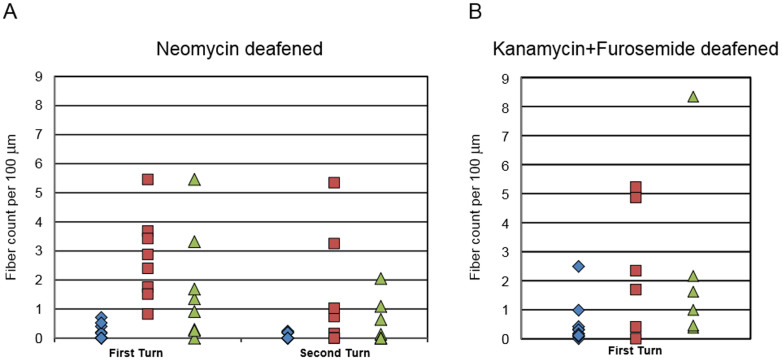
Comparison of Peripheral Auditory Fiber Re-growth Between Experimental Groups. In neomycin deafened ears, either AAV.*Ntf3* or AAV.*BDNF* led to a significantly greater number of fibers in the basal turn as compared to deafened, un-injected control ears (A). However, the significant effect of neurotrophin over-expression on fiber re-growth faded in the second cochlear turn. Inoculation with either AAV.*Ntf3* or AAV.*BDNF* led to greater fiber re-growth in the basal turns of systemically deafened ears, though only NT-3 led to significantly greater fiber re-growth (B). Blue = control; red = NT-3; green = BDNF.

Quantification of the PAFs in neomycin-deafened ears showed that AAV-mediated over-expression of either neurotrophin (BDNF or NT-3) resulted in significantly more PAFs in the basal turn as compared to deafened control (no neurotrophin vector) cochleae (Fig. 3A). Ears injected with the NT-3 vector had 2.74 PAFs (standard error of mean ± 0.52) per 100 microns and ears that received the BDNF vector had 1.66 ± 0.71 PAFs, which were both significantly different from the 0.14 ± 0.06 PAF observed in the control ears (p < 0.05). The effects of the neurotrophin gene transfer dissipated greatly in most ears by the second turn, and there was no significant difference overall in the number of PAFs in the second turn of neomycin-deafened ears when comparing those receiving neurotrophin vector and those that were not. However, a few ears showed substantially more peripheral fiber counts than any of the control ears. There was no significant difference between the effects of NT-3 and BDNF on the neomycin deafened ears (p = 0.22 and 0.18 for the basal and second turns, respectively). In K&F deafened ears (Fig. 3B), assessment in the basal turn showed significant PAF regrowth induced by NT-3 (2.42 ± 0.90 vs 0.46 ± 0.30, p < 0.05).

### The relationship between presence of supporting cells and PAF re-growth

**Figure 4:**
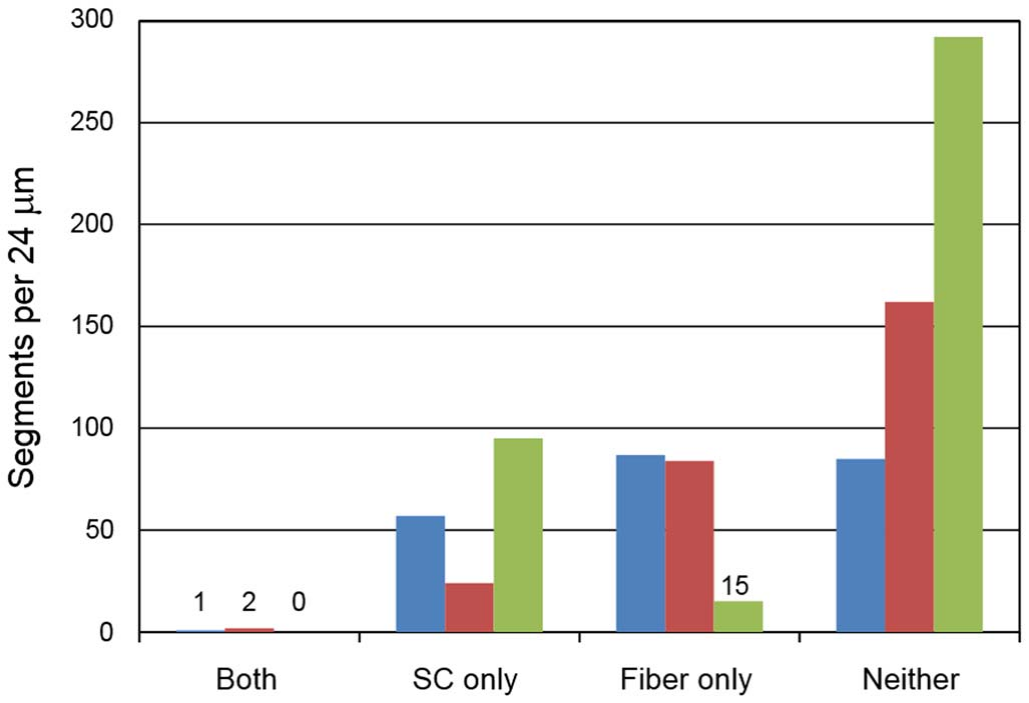
Correlation between Presence of Peripheral Auditory Fibers and Supporting Cells. The presence of PAFs in regions with surviving supporting cells was extremely rare in systemically deafened ears. More commonly, only supporting cells, only PAFs or neither were seen within a given radial segment of the organ of Corti. Blue = control; red = NT-3; green = BDNF.

In the course of examining the whole-mounted tissues from the systemically deafened ears, a pattern began to emerge with PAFs tending to be present in regions where supporting cells were absent. Formal quantitative analysis (as outlined in the methods section and illustrated in Suppl. Fig. 2) revealed a significant negative correlation between the presence of supporting cells and PAFs for each of the two neurotrophin vectors (NT-3 p < 0.00001, BDNF p = 0.0058) (Fig. 4). A total of only 3 segments, 2 in NT-3 ears and 1 in an BDNF ear, had both differentiated supporting cells and PAFs. In contrast, the vast majority of segments had only supporting cells (NT-3, 57 segments; BDNF, 24 segments), only PAFs (NT-3, 87 segments; BDNF, 84 segments) or neither supporting cells nor PAFs (NT-3, 85 segments; BDNF, 162 segments). A similar correlation analysis was not indicated in the neomycin-deafened ears as they almost universally had flat epithelium in the basal and most of the second cochlear turns. We concluded that the survival of Deiters and pillar cells was negatively correlated with neurotrophin-induced sprouting of PAFs.

### The effects of neurotrophin over-expression on SGN survival

**Figure 5:**
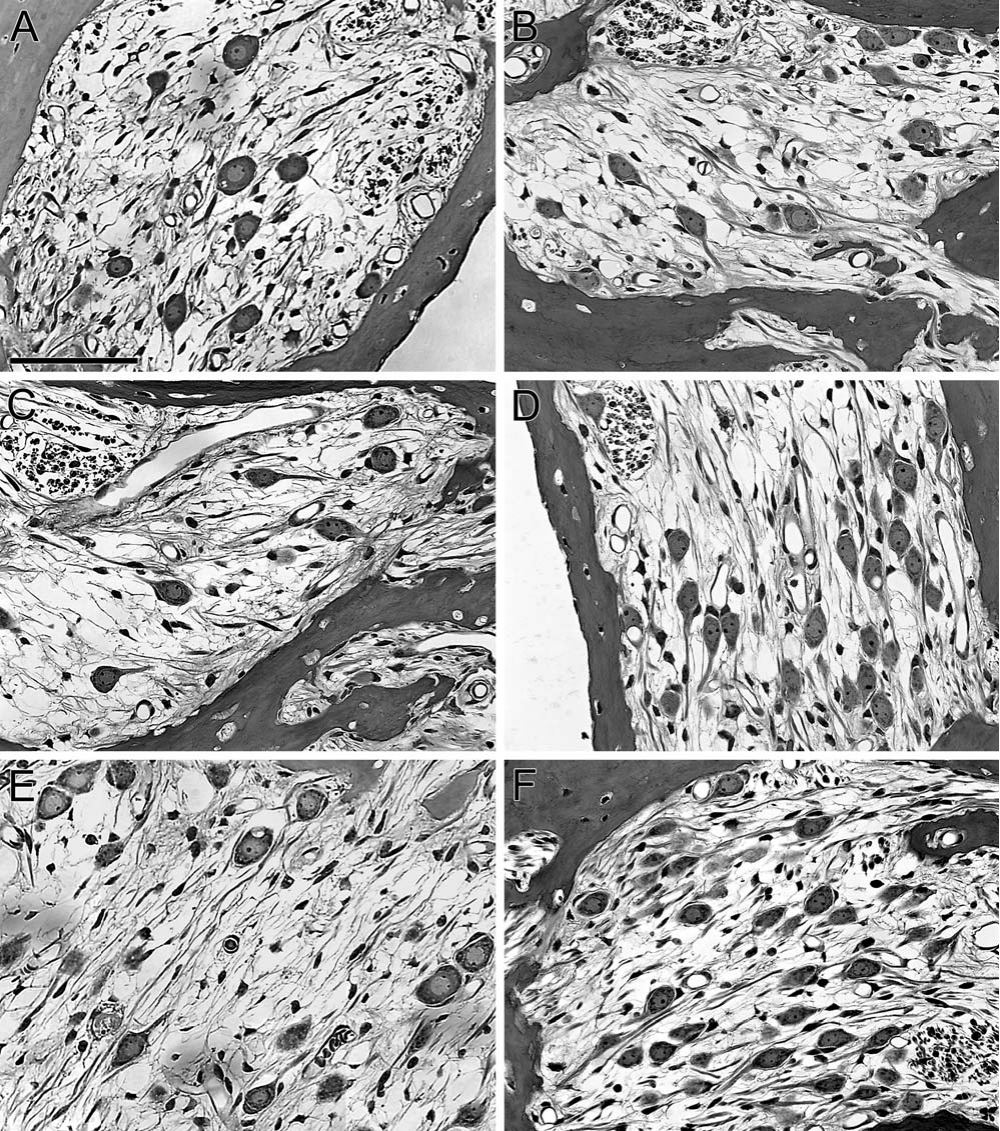
Spiral Ganglion Survival with Neurotrophin Over-Expression. Representative sections from cochleae deafened with neomycin (A, C and E) or K&F (B, D and F) with no further procedure (A, B), AAV.*Ntf3* injection (C, D) or AAV.*BDNF* injection (E, F). Neomycin deafened ears injected with AAV.*BDNF* (E) had significantly greater SGN survival than neomycin deafened, un-injected control (A) or NT-3 ears (C). In systemically deafened ears, neither BDNF (F) nor NT-3 (D) led to consistently improved SGN survival as compared to un-injected control ears (B), likely related to the less severe lesion induced by the systemic deafening itself. Scale bar = 50 μm.

Neurotrophin vector injection led to greater SGN survival in the basal turn (lower and upper profiles) of deafened ears. Qualitative examination of the cross sections through Rosenthal's canal (Fig. 5) revealed that the surviving SGNs had well defined nuclei and nucleoli and a typical spherical shape in both neomycin deafened ears (A, C and E) and in K&F deafened ears (B, D and F), whether they received neurotrophin expressing vectors or not. Pyknotic neurons or cells undergoing apoptosis were not typically seen in these sections, suggesting that the neurons present at this stage, 3 months after the deafening, were stable and viable, at least based on their morphology.

**Figure 6:**
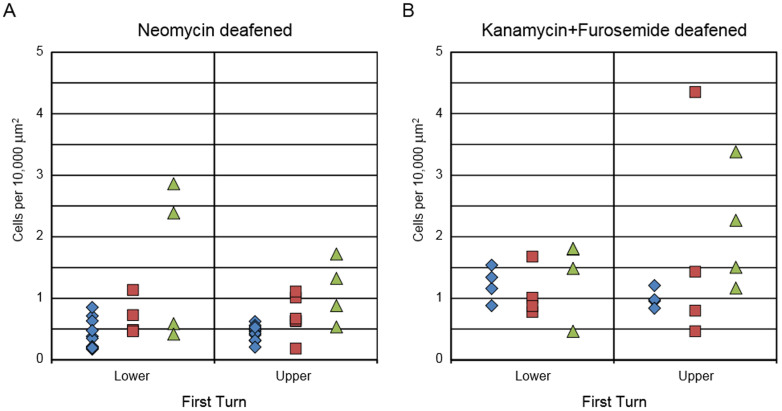
Enhancement of Spiral Ganglion Neuron Survival is Function of Neurotrophin and Method of Deafening. Neomycin deafened ears injected with AAV.*BDNF* had significantly more SGNs in the basal turn than AAV.*Ntf3* injected or un-injected control ears, especially in the lower part of the turn (A). Some ears that received NT-3 also had more SGNs than un-injected controls; however, the effects of NT-3 were smaller and the difference between means did not reach significance. Neither neurotrophin led to a significant increase in SGN survival in ears deafened with kanamycin and furosemide, although some individuals did have substantially higher SGN survival in the upper part of the first turn (B). Blue = control; red = NT-3; green = BDNF.

Evaluation of the neomycin deafened ears revealed a significant difference in SGN survival between neurotrophin vector groups (p = 0.003, Fig. 6A). SGN density in eight contralateral ears was not significantly different from four ears receiving the empty vector (0.61 ± 0.21 SGN/10,000 μm^2^ vs. 0.45 ± 0.11, p = 0.46), and so the data from the contralateral (no vector injection) ears and the AAV.Empty ears were pooled in subsequent analyses to increase statistical power of tests for effects of vectors with neurotrophin gene inserts. In the lower basal turn, neomycin deafened ears that received the BDNF vector averaged SGN 1.72 ± 0.34, more than twice as many as in NT-3 ears (0.70 ± 0.16) and more than three times as many as the pooled AAV.Empty controls and un-injected contralateral ears (0.50 ± 0.09). Taken together, these results indicate that the rescue effect on SGNs in the deaf ears receiving AAV.*BDNF* can only be attributed to the neurotrophin gene insert.

The effect of AAV.*BDNF* on SGN survival was less pronounced in the upper basal turn; the effect of AAV.*Ntf3* did not demonstrate a similar difference between upper and lower parts of the first turn (BDNF, 1.00 ± 0.18; NT-3, 0.71 ± 0.13; control, 0.49 ± 0.04). Similar proportions of ears in the two neurotrophin groups had more SGN than controls, but the magnitude of improvement tended to be less in the NT-3 group ears. Because the effect of BDNF declined sharply with distance, the neurotrophin groups were significantly different only in the lower section of the basal turn.

In systemically deafened ears, neither neurotrophin led to consistently improved SGN survival compared to control ears that received no neurotrophin vector (Fig. 6B). Some systemically deafened ear that received the neurotrophin vector did demonstrate greater SGN survival than that seen in control ears, but this response was quite variable and many ears demonstrated equivalent survival between the neurotrophin vector injected and non-injected control ears. The number of systemically deafened ears that had improved SGN survival with neurotrophin vector injection was not significantly different between the NT-3 and BDNF groups of ears.

## Discussion

Both deafening methods, local intracochlear infusion of neomycin and systemic administration of K&F, were effective in inducing a hearing loss, as assessed by the Preyer reflex and loss of hair cells. However, the deafening methods had different effects on the cochlea, with the intracochlear infusion of neomycin causing a more severe lesion. Intracochlear infusion of neomycin led to a complete loss of hair cells throughout all cochlear turns and a loss of Deiters and pillar cells, leaving a flat epithelium throughout the basal turn. In contrast, systemic administration of K&F led to a complete loss of hair cells only in the basal turn with a patchy replacement of supporting cells with flat epithelium in that turn. Thus, these two methods of deafening guinea pig ears produce a range of conditions similar to that seen in humans with hearing loss, and were chosen to model this range of inner ear lesions.

We determined that neurotrophin over-expression with either AAV.*Ntf3* or AAV.*BDNF* enhanced PAF re-growth in previously deafened ears. The effectiveness of the neurotrophin over-expression outcome did not differ between the neurotrophins, but did appear to be influenced by the deafening method, with greater PAF re-growth when gene transfer followed neomycin deafening than when it followed systemic (K&F) deafening. This influence of deafening method may be due to the presence of many patches of surviving Deiters cells and pillar cells in the systemically-deafened ears, which seemed to inhibit neurite regrowth. The effect of neurotrophin over-expression on SGN survival after deafening also was affected by deafening method, with only BDNF significantly increasing SGN survival in neomycin deafened ears, and neither neurotrophin increasing SGN survival in systemically deafened ears.

An unexpected finding from this study was the inverse relationship between PAF re-growth and the presence of supporting cells in the systemically deafened, neurotrophin-receiving ears. In these ears, PAFs were rarely found in regions of surviving supporting cells. The reason for this negative correlation is unclear, as PAFs demonstrate an ability to weave under or between cells of the flat epithelium. The negative correlation between surviving supporting cells and re-grown PAFs is a striking contrast to previous findings demonstrating a positive correlation between survival of pillar cells and survival of SGNs and their PAF. For instance, a prior study demonstrated that following an ototoxic insult, neuronal structures were better preserved in regions with intact supporting cells^18^. The correlation between supporting cell and auditory neuron survival has particularly been noted in the apical turns of deafened cochleae^18^,^19^. These data suggested that supporting cells provided trophic support to the auditory neurons. The difference between our results and those of previous studies may indicate that over-expression of neurotrophins early after the deafening changes the pattern of degenerative and/or regenerative events in the cochlea. The lack of PAF extending to supporting cells in the basal turn at 3 months after K&F deafening suggests that in our model system, the trophic support provided by Deiters and pillar cells is absent, or that supporting cells pose a mechanical or chemical barrier to re-growth of PAFs.

Our results do not conflict with hypotheses that greater supporting cell and auditory neuronal survival in the apical turns as compared to the basal turns may be due to an unexplained apex-base gradient. Findings from our current study lend support to the theory that more supporting cells and auditory neurons survive in the apical turns due to an apex-base gradient as the systemically deafened ears had significantly greater hair cell, supporting cell and auditory neuronal survival in the apical turns. In addition, our results reinforce the hypothesis that supplementary neurotrophins are needed for long-term PAF survival in the absence of hair cells to stimulate persistence of the neurotrophin feedback loop.

Differences in the effects of NT-3 and BDNF have important implications for understanding the functions of these neurotrophins and for assessing their suitability as therapeutic agents. Previous studies have suggested these neurotrophins have different targets during inner ear development^7^,^20^. Our study suggests they may also have different roles in the maintenance of neuronal tissue in adults exposed to ototoxic drugs. Both neurotrophins elicited more than 10× as many PAF as in control ears, and NT-3 induced 2× as many as BDNF. In contrast, SGN survival was increased over controls by only a factor of 3 using BDNF, and by less than half that using NT-3. Thus BDNF had a greater effect enhancing SGN survival while NT-3 had a greater effect eliciting PAF re-growth from surviving cells. While it may not be possible to save all SGNs in a severely deafened cochlea, it is likely that partial survival, along with regeneration of PAF, would improve CI outcomes and potentially provide a healthy neural substrate for regenerated hair cells. For this purpose, it is possible that a combined over-expression of both NT-3 and BDNF will comprise the optimal outcome for enhancing the cochlear neural substrate.

Neurotrophins have been delivered to the inner ear of animal models in a variety of ways, including through the use of mini-osmotic pumps or one of several viral vectors. Osmotic pumps were not used in this study because their clinical applicability is limited by non-specific delivery of the therapeutic agent into the perilymphatic space and by the need to periodically refill the reservoir with the therapeutic agent. In this study, we tested the effects of inoculation with AAV expressing neurotrophins primarily because of their documented long-term gene expression following a single inoculation and lack of side effects, as determined in other systems^17^. Indeed, the use of AAVs did not appear to elicit a major inflammatory response, as could be determined by clear cochlear fluids and survival of some apical hair cells and supporting cells. However, ELISA assays of the perilymph from animals euthanized at 3 months determined that most BDNF receiving ears did not exhibit elevated levels of the neurotrophin in perilymph and less than half of the NT-3 ears had elevated neurotrophin in the fluid. This raises important questions related to AAV-mediated gene delivery via the perilymph. The fact that at 2 different time points; 3 weeks and three months following inoculation, there was increased PAF survival as compared to deafened ears that were not inoculated with a viral vector carrying a neurotrophin gene suggests that at earlier times the viral vector was actively supplying neurotrophins, a finding that is corroborated by our in vitro data. However, the induced response to the AAV vector at 3 months was not as robust, both in terms of the number and the distribution of re-grown PAFs, as compared to the response to an adenoviral vector at less than one month following inoculation^21^. It is known that adenoviral vectors tend to induce more robust but shorter term expression of their gene inserts^22^,^23^ than do AAV vectors^17^,^24^. We speculate that the effect of the AAV vector diminished gradually over time, possibly due to death of the transfected cells. Like all fibroblastic cells, mesothelial cells are likely to have slow turnover, which could possibly be accelerated by the presence of the AAV vector in these cells. Therefore, differences in proportions of transfected cell types could account for the differences in neurotrophin levels observed at 3 months. Because epithelial cells that line the scala media are largely quiescent and likely long living cells, they may be a better target for long term gene expression via gene transfer.

Differences in effectiveness between basal and apical turns were observed for both deafening method and both neurotrophins. In all cases, the largest effects were seen in the lower basal turn. The most severe lesions after deafening, and the greatest positive effects of the neurotrophin over-expression procedure, were primarily seen in the basal turn of the cochleae. Intracochlear fluid dynamics may explain this spatial distribution for the neomycin deafening and for the neurotrophin vector injection outcomes, as these procedures were both delivered by an infusion into the basal cochlear turn^25^,^26^. However, other mechanisms are needed to explain this effect in systemic deafening.

In summary, both AAV.*Ntf3* and AAV.*BDNF* led to significantly greater PAF re-growth in the basal turns of previously deafened adult guinea pig inner ears. There was no significant difference between the two neurotrophins in the number of PAFs present in the basal neuroepithelium, though on average there were more PAFs in ears that received the NT-3 vector as compared to ears injected with the BDNF vector. In contrast, only inoculation of AAV.*BDNF* led to significantly greater SGN survival in neomycin-deafened ears. Systemic deafening led to a less severe cochlear lesion overall, and it did not lead to a significant decline in SGN survival. Systemically deafened ears receiving either neurotrophin were devoid of PAFs in regions of surviving supporting cells, suggesting that the state of differentiation of the non-sensory epithelium influences PAF regrowth.

## Methods

### Animal Subjects

Pigmented, specific pathogen free guinea pigs of both genders served as the animal model for this study. Animals were one to two months old and weighed between 250 and 500 grams at the onset of the procedures. This study was approved by and performed in accordance with guidelines established by the University of Michigan's University Committee on the Use and Care of Animals. A total of 46 animals were included in this study. The experimental animals were divided into eight groups based on the deafening method used (systemic K&F or bilateral intracochlear neomycin), the neurotrophin vectors administered (AAV.*Ntf3* or AAV.*BDNF*), and the histological method (whole mounts or modiolar cross sections) (Table 1). Animals were euthanized 3 months following inoculation and then processed for histology. The majority of animals were processed for whole mounts of the organ of Corti for the purpose of quantifying PAF re-growth. A smaller subset of cochleae from each group was embedded in JB4 for plastic sectioning to quantify SGN survival. Additional control animal groups received AAV.Empty (N = 4, sacrificed after 3 months and processed for plastic sections and SGN counts), or inoculation of AAV.*BDNF* (N = 3, sacrificed after 3 weeks and processed for whole-mounts stained for neurofilaments). All animals were deafened bilaterally to provide an internal control (deafened, un-injected) ear, and then received the AAV vector inoculation unilaterally, in the left ear.

### Surgical Procedures

We selected two methods for deafening. A systemic insult was used to induce loss of hair cells with a less severe lesion to the supporting cells. Destruction of hair cells while leaving some intact supporting cells was accomplished by K&F. The second method involved an infusion of neomycin into the perilymph. This was used to induce a more severe lesion that eliminated not only hair cells but also supporting cells, leaving a flat epithelium.

Prior to each surgical procedure, the animals were anesthetized with an intramuscular injection of xylazine (10 mg/kg) and ketamine (40 mg/kg). All animals were deafened on Day 0 of the protocol. Neomycin deafened animals underwent bilateral infusion of 10 μL of 5% neomycin (weight of active drug/volume) into the scala tympani through a basal cochleostomy. A relatively large volume of 10 μL was utilized to ensure perfusion of neomycin throughout the cochlear turns, and diffusion of neomycin through the cerebrospinal fluid to the contralateral ear was considered inconsequential given the goal of bilateral deafening. The basal turn of the cochlea was exposed through a post-auricular incision and opening of the temporal bulla, and then the neomycin was infused using a motorized pump, glass Hamilton syringe and micro-cannula. The cochleostomy was then sealed with Durelon carboxylate dental cement (3 M ESPE, Seefeld, Germany). Systemically deafened animals were given an intravenous dose of furosemide (100 mg/kg) after surgical exposure and cannulation of the internal jugular vein, immediately followed by administration of kanamycin (400 mg/kg) by a subcutaneous injection. Vector inoculations were performed a week later to allow animals to recover from the surgery and to maximize hair cell elimination.

One week following deafening, on Day 7 of the protocol, all animals underwent unilateral inoculation with a viral vector (AAV.Empty, AAV.*Ntf3* or AAV.*BDNF*) delivered through a basal cochleostomy. Similar to the neomycin deafening procedure, the basal cochlea was exposed through a post auricular incision and opening of the temporal bulla. A cochleostomy was made into the scala tympani near the round window using a sharp pick. In those ears that had previously undergone deafening by an intracochlear infusion of neomycin, the same cochleostomy was reopened for inoculation with the viral vector. A total volume of 5 μL of the viral vector was then administered at 1 μL/minute using a motorized pump, Hamilton glass syringe and micro-cannula. Specifically, the viral vectors (gift from the Adriana Di Polo, University of Montreal) were adeno-associated virus type II with a CBA promoter carrying the mouse gene insert for either *Ntf3* (PFU/mL = 1.45 × 10^12^) or *BDNF* (PFU/mL = 2.35 × 10^11^). The cochleostomy was sealed at the end of the procedure using Durelon carboxylate dental cement. Three months following inoculation all animals were anesthetized (as described above) and then euthanized as specified below for each analysis group.

### Peripheral Auditory Fiber Quantification

Following anesthetization and decapitation, the temporal bones were extracted and locally perfused with 4% paraformaldehyde (PFA). The temporal bones were fixed in the 4% PFA for 1 hour and then stored in phosphate buffered saline (PBS) until further processing could be undertaken. The otic capsule bone was peeled away to expose the modiolus and soft tissues in the cochlea. Tissues were then stained with antibody specific for neurofilament 200 kDa and with phalloidin to visualize F-actin. Tissues were first permeabilized in 0.3% Triton X-100 for 10 minutes, and then incubated in 5% normal goat serum for 30 minutes to block against non-specific binding of the secondary antibody. The tissues were then incubated in anti-neurofilament 200 kDa rabbit polyclonal antibody diluted to 1:200 in PBS for one hour, and then rinsed with PBS twice. Samples were incubated next in goat anti-rabbit rhodamine (1:200) secondary antibody for thirty minutes. Samples were then counterstained with Alexa phalloidin 488 for F-actin (1:200) for one hour. After rinsing with PBS, tissues were whole mounted, cover-slipped and observed with a Leica DMRB epi-fluorescence microscope (Leica, Eaton, PA, USA).

For each ear, the full length of the dissected auditory epithelium was examined. Images were taken using a 40× objective lens from all segments of the auditory epithelium that were not extensively damaged in the course of dissection. Of all the images taken from each ear, five images were selected blindly and randomly to undergo further quantitative analysis. Images from the first and second turns were used in analysis of the neomycin-deafened ears, whereas images from the first turn only were used in analysis of the systemically deafened ears. Images from the first turn only were used from the systemically deafened ears as these ears displayed inner hair cell survival beginning in the second turn, which would have confounded the PAF survival results. Neuronal fibers extending more than 50 μm radially from the habenula perforata were counted in each image^21^, and the number of fibers was summed to generate a single number for each cochlea. The length of the auditory epithelium captured by one image was measured longitudinally along the habenula perforata, and the density of neuronal fibers as a function of epithelial length was calculated.

The number of PAFs was assessed and compared between the following groups: neomycin-deafened, AAV.*Ntf3* injected (n = 8); neomycin-deafened, AAV.*BDNF* injected (n = 8); systemically deafened, AAV.*Ntf3* injected (n = 6); systemically deafened, AAV.*BDNF* injected (n = 6); and contralateral controls for neomycin-deafened (n = 14) and systemically deafened (n = 11) ears. Two control ears from the neomycin-deafened group and one control ear from the systemically-deafened group could not be included in the analysis due to significant ossification in the basal turn, precluding adequate dissection. Two-way analysis of variance (ANOVA) was used to evaluate whether a significant difference existed among neurotrophin vector groups, followed by a post-hoc two-tailed Student's *t*-test to evaluate statistical significance between neurotrophin group pairs. The sequential Bonferroni criterion was used to adjust for multiple comparisons, with a table-wide significance level of 0.05.

### Correlation of Peripheral Auditory Fiber and Supporting Cell Presence

The relationship between the presence of surviving supporting cells (Deiters cells and pillar cells) and PAFs in systemically deafened ears was also assessed. A second set of five images was selected at random from the first turns of cochleae systemically deafened and neurotrophin vector injected. For each image, the auditory epithelium was divided into radial segments of 24 μm each measured longitudinally along the habenula perforata (Suppl. Fig. 2). Each radial segment was evaluated for the presence of supporting cells and neuronal fibers. The number of radial segments with only supporting cells, only PAFs, both supporting cells and PAFs, or neither was counted to generate four numbers per cochlea. These numbers were analyzed in 2 × 2 contingency tables to test whether the distribution of PAFs was contingent upon the distribution of supporting cells. Deviation from expectations under a random distribution, indicating a significant correlation between the presence of supporting cells and PAFs, was evaluated using a chi-squared distribution.

### Analysis of neurotrophin concentration in conditioned media

Guinea pig fibroblasts were seeded into 6-well tissue culture plates, 120,000 cells/well. Twenty four hours later, culture media was removed and 1 ml of fresh media was added. 1 μl of AAV.*BDNF* or AAV.*Ntf3* virus was added per well. Cells were exposed to the AAV for 4 hours then media was removed and 1.5 ml of fresh media was added. Another 1 ml of media was added 3 days later. Media was collected 7 days later and stored at −20° until analyzed. Three wells each were cultured with the following virus vectors: AAV2.*GFP* UF11 (lot# N3619), AAV2.*CBA.BDNF* (lot# 3618) and AAV2.*smNtf3* (lot# 2720). Three additional control wells received no virus. The viral vectors used for the culture experiments were from the same source and batch as those used for the in vivo experiments.

Concentrations of neurotrophin in media samples were evaluated using Millipore ChemiKine BDNF kit and the IBL-America NT-3 kit, following the manufacturers' instructions. The manufacturer supplied neurotrophin solution, diluted per instructions was used as a positive control (highest prepared concentration) and to set standard curve to infer concentrations of unknowns (optical densities compared to serial dilutions). For each analysis, a standard curve with high correlation between concentration and neurotrophin concentration (r^2^ > 0.9) was obtained, and the concentrations of the media samples were extrapolated from that curve. Student's t-test was used to compare concentrations between samples receiving the vector carrying the neurotrophin gene and those receiving GFP/control vectors or no vector.

### Analysis of neurotrophin concentration in perilymph

To assess neurotrophin concentration in perilymph, the fluid was extracted from the cochlea of the anesthetized animal immediately prior to decapitation. Guinea pigs were anesthetized and the surgical site was reopened. A Hamilton syringes with the micro cannula was inserted into the prior cochleostomy site to extract 3–5 μl of perilymph. This procedure minimizes the risk of contamination with cerebrospinal fluid but does not prevent it altogether. The extracted fluid was stored at −80°C until the ELISA was performed.

Neurotrophin concentrations were assayed by ELISA using the Millipore ChemiKine kit for BDNF, or the IBL-America kit for NT-3. Due to the small sample volumes, 10 μl of the appropriate sample diluent was added to each sample to provide sufficient total volume to cover the bottom of each well. In addition to the manufacturer's suggested dilution of the reconstituted protein standard to construct a standard curve, a 5 μl sample of reconstituted standard (prior to further dilution) was processed identically to the samples, to provide additional confirmation of the concentrations inferred from these small volumes. Using this protocol it was possible to confirm that protein concentrations could be measured from such small volumes, but with an elevated detection threshold of 2–3 times the manufacturer's estimated threshold.

### Spiral Ganglion Neuron Quantification

A subset of cochleae from each neurotrophin group was analyzed by light microscopy of the modiolar cross sections in order to count the number of SGNs within Rosenthal's canal. In addition to the two neurotrophin groups, a group of deaf cochleae (neomycin) injected with AAV.Empty was also prepared for SGN counts. Following anesthetization, these animals were first perfused with phosphate buffered saline. Then, for initial fixation, the animals were perfused systemically with 2% glutaraldehyde in 0.15 mol/L cacodylate buffer. The temporal bones were removed from the skull, the cochleae exposed, and the otic capsule and windows opened to enhance fixative access. The cochleae were immersed in the same solution for 2 hours. Cochleae were then immersed in 5% EDTA containing 0.5% glutaraldehyde for 10–14 days to decalcify the bone. The tissue was then dehydrated with ethanol, embedded in JB-4 resin and sectioned on a microtome using glass knives. 3 μm sections were obtained at the mid-modiolar plane, providing six distinctive Rosenthal's canal cross-sections within each microtome section. Each section was then stained with toluidine blue, covered and assessed under light microscopy.

To determine the number of morphologically intact SGNs within each turn, images were acquired from non-sequential plastic sections at least 9 μm apart to prevent double-counting the same SGN across different sections. Only cells with clear nuclei and nucleoli were counted. SGN survival in each deafening and neurotrophin group was tested for significance with two-way ANOVA.

## Supplementary Material

Supplementary Information Dataset 1-2

## Figures and Tables

**Table 1:** Animal neurotrophin groups. Guinea pigs were divided into four groups according to the deafening method (systemic kanamycin and furosemide, or bilateral intracochlear neomycin) and neurotrophin over-expression (NT-3 or BDNF) administered. The majority of animals in each group were processed for whole mounts of the organ of Corti in order to assess peripheral auditory fiber re-growth. A minority of animals from each group was processed for JB4 plastic sectioning to assess spiral ganglion neuron survival

	Deafening Method	Neurotrophin	N	Total per Group
**PAF (Whole Mounts)**	Local Neomycin	NT-3	8	28
		BDNF	8	
	Systemic K&F	NT-3	6	
		BDNF	6	
**SGN (Modiolar Cross Sections)**	Local Neomycin	NT-3	6	18
		BDNF	4	
	Systemic K&F	NT-3	4	
		BDNF	4	
