# Supplementary material for: Differential Effects of AAV.BDNF and AAV.Ntf3 in the Deafened Adult Guinea Pig Ear
Source: Sci Rep. 2015 Mar 2;5:8619. doi: 10.1038/srep08619 (PMC4649680; doi:10.1038/srep08619)

**Supplemental Material Cover Page**

**For paper:**

**Differential Effects of AAV.*BDNF* and AAV.*Ntf3* in the Deafened Adult Guinea Pig Ear**

**SREP-14-02646D**

**Author List: Cameron L. Budenz, Hiu Tung Wong, Donald L. Swiderski, Seiji B. Shibata, Bryan E. Pfingst, *Yehoash Raphael**

**
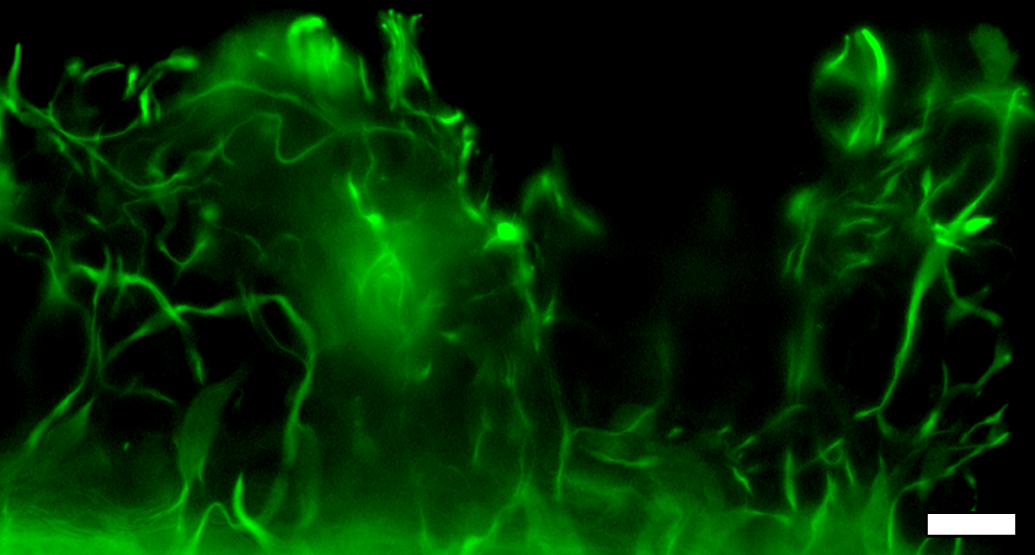
**



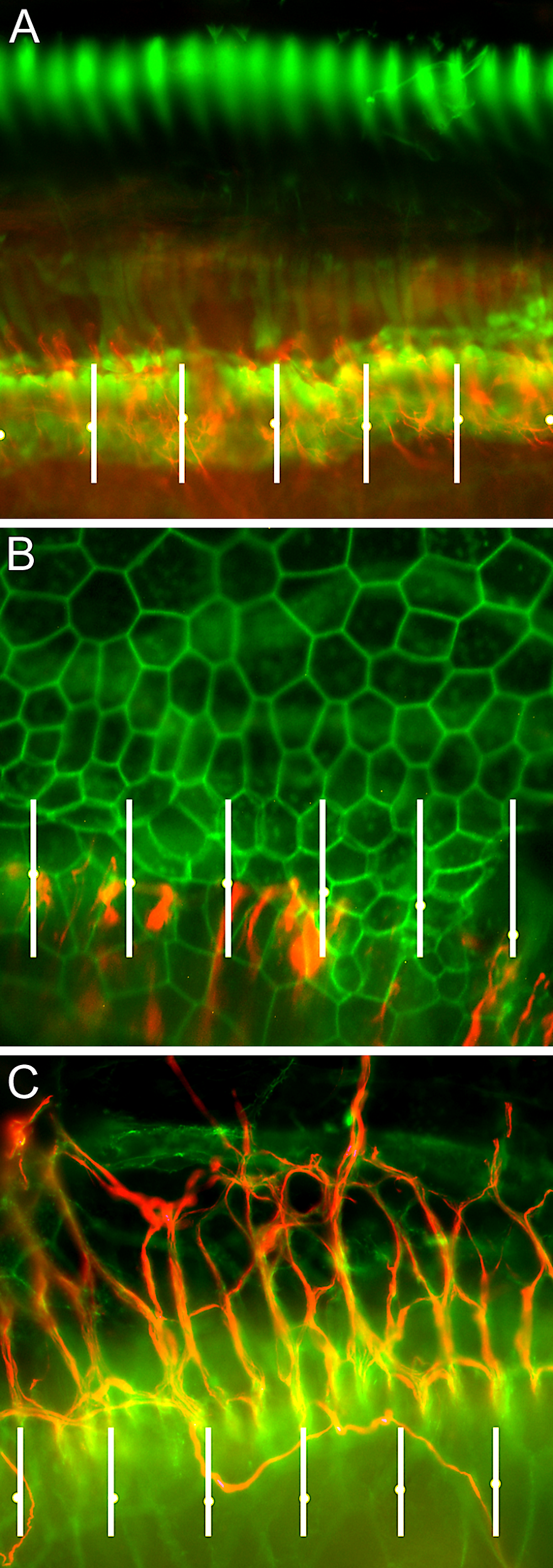

Supplement: Supplementary Information — Dataset 1-2 [file srep08619-s1.docx]
